# Comparing mechanism-based and machine learning models for predicting the effects of glucose accessibility on tumor cell proliferation

**DOI:** 10.1038/s41598-023-37238-2

**Published:** 2023-06-27

**Authors:** Jianchen Yang, Jack Virostko, Junyan Liu, Angela M. Jarrett, David A. Hormuth, Thomas E. Yankeelov

**Affiliations:** 1 Department of Biomedical Engineering, The University of Texas at Austin, 107 W. Dean Keaton, BME Building, 1 University Station, C0800, Austin, TX 78712 USA; 2 Department of Diagnostic Medicine, The University of Texas at Austin, Austin, TX 78712 USA; 3 Department of Oncology, The University of Texas at Austin, Austin, TX 78712 USA; 4 Oden Institute for Computational Engineering and Sciences, The University of Texas at Austin, Austin, TX 78712 USA; 5 Livestrong Cancer Institutes, The University of Texas at Austin, Austin, TX 78712 USA; 6 Departments of Imaging Physics, The University of Texas MD Anderson Cancer Center, Houston, TX 77030 USA

**Keywords:** Computational models, Cancer, Biomedical engineering

## Abstract

Glucose plays a central role in tumor metabolism and development and is a target for novel therapeutics. To characterize the response of cancer cells to blockade of glucose uptake, we collected time-resolved microscopy data to track the growth of MDA-MB-231 breast cancer cells. We then developed a mechanism-based, mathematical model to predict how a glucose transporter (GLUT1) inhibitor (Cytochalasin B) influences the growth of the MDA-MB-231 cells by limiting access to glucose. The model includes a parameter describing dose dependent inhibition to quantify both the total glucose level in the system and the glucose level accessible to the tumor cells. Four common machine learning models were also used to predict tumor cell growth. Both the mechanism-based and machine learning models were trained and validated, and the prediction error was evaluated by the coefficient of determination (*R*^2^). The random forest model provided the highest accuracy predicting cell dynamics (*R*^2^ = 0.92), followed by the decision tree (*R*^2^ = 0.89), *k*-nearest-neighbor regression (*R*^2^ = 0.84), mechanism-based (*R*^2^ = 0.77), and linear regression model (*R*^2^ = 0.69). Thus, the mechanism-based model has a predictive capability comparable to machine learning models with the added benefit of elucidating biological mechanisms.

## Introduction

The dysregulation of cellular energetics is one of the hallmarks of cancer^1^. The altered metabolic activities (e.g., consumption and utilization of glucose by tumor cells), varying between aerobic glycolysis and oxidative phosphorylation, demonstrates tumor cell adaptation to the microenvironment to fulfill its need for energy and materials for biosynthesis^1^. Therefore, glucose plays a central role in driving tumor metabolism and development. Tumor cell proliferation in response to the availability of glucose has become an intense area of investigation to determine the underlying mechanisms of tumor cell metabolism and development, as well as identify potential therapeutic targets for altering the uptake and consumption of glucose. Targets of interest include (for example) glucose transporters^2^, lactate transporters^3^, and the enzymes hexokinase and pyruvate kinase of the glycolytic pathway^4,5^. In the present study, we are particularly interested in the glucose transporters as they directly influence intracellular glucose concentration which is the starting material for both glycolysis and oxidative phosphorylation.

Mechanism-based mathematical models are playing an increasingly important role in cancer biology and oncology, as they can describe observations from experiments and clinical trials, provide predictions of tumor development and response to therapy, guide experimental design, identify new hypotheses, and optimize treatment delivery^6–15^. There have been a number of attempts to construct models to simulate and understand the development of tumor subpopulations, the dynamics of extra- and intra-cellular nutrient concentrations, nutrient demands during different stages of the cell cycle, and tumor-environment interactions. These phenomena have been modelled with various approaches including ordinary differential equations (ODEs)^16^, partial differential equations (PDEs)^17–21^, agent-based models^22^, and game theoretical models^22–25^. While these approaches have provided theoretical insights into tumor metabolism via simulations, few direct calibrations of the models to experimental data have been performed, leaving them unvalidated and challenging to use for predictions. To begin to address this need, we recently^26^ simplified the model developed by Mendoza-Juez et al.^16^ to a smaller system with a limited number of (four) parameters that we were able to calibrate with time-resolved microscopy data, and then predict tumor cell growth by running the model forward in time. However, that model does not account for nutrient perturbations resulting from the application of nutrient transporter inhibitors; indeed, only a few efforts to account for such interventions have been attempted.

Cui et al. presented an early mathematical investigation on the effect of a general inhibitor (e.g., immune system response, anti-cancer drugs, or radiation therapy) on tumor growth^27^. A spherically symmetric tumor that included both nutrient species and inhibitor species was modeled and asymptotic behavior was theoretically determined. The results of the analysis indicated that reducing the nutrient level and increasing inhibitor level presented a similar effect on the tumor’s final size. Many subsequent theoretical papers were built on this foundational study on the effects of a nutrient inhibitor on tumor dynamics. Linking such modeling insights to experimental data is the next critical next step.

Roy and Finley et al. developed a system of ODEs to describe a metabolic network comprised of 53 enzymatic reactions involving 46 metabolites^28^. The model included 71 reaction rates to be calibrated within 46 ODEs with another 301 parameters assigned from the literature. They employed the model to predict, in silico, the effect of metabolic perturbations, including varying glucose availability or knockdown of glucose transporters, on cell proliferation. This allowed them to theoretically explore all the options of performing enzyme-encoding gene knockdown, inhibiting metabolism, and perturbating extracellular conditions to identify combinations that could inhibit cellular proliferation. The authors noted that exploring the space experimentally is prohibitive and speaks to the utility of this kind of modeling to identify testable hypotheses.

Complimentary to mathematical models that seek to explicitly incorporate the biology of the phenomena under investigation are data-driven models that seek to extract hidden patterns within large quantities of data. Without requiring specific knowledge about the underlying biology, machine learning approaches can be applied when only incomplete or limited knowledge is available for a study. Many efforts have been made to study cancer metabolism applying either unsupervised^29^ or supervised learning^30–32^ on diverse data sources. For example, Eyassu and Angione performed principal component analysis (PCA) on data that described metabolites flowing between glycolysis and the tricarboxylic acid (TCA) cycle from different conditions to identify key reactions for the study of the pyruvate dehydrogenase (PDH), an essential component involved in reprogramming of cancer metabolism^29^. Kim et al. built a random forest classifier to predict breast cancer metastasis using RNA-seq data with high accuracy^31^. Katzir et al*.* integrated multi-omics (transcriptomics, proteomics, phosphoproteomics, and fluxomics) data to predict which enzymes and pathways are regulated within the MCF-7 breast cancer cells via support vector machine (SVM) classification^30^. Gomez et al*.* combined several feature selection approaches and classifiers from machine learning on FDG-PET/CT (i.e., fluorodeoxyglucose positron emission tomography/x-ray computed tomography) data to predict metabolism in response to treatment targeting metastatic breast cancer^32^. These examples indicate that machine learning approaches can provide insights when a mechanistic understanding of cancer metabolism is not known.

In this contribution, we monitored the temporal dynamics of both the growth and death of breast cancer cells in vitro utilizing time-resolved microscopy, in a series of experiments involving different initial conditions (glucose concentrations and confluences), with or without applying an inhibitor of glucose uptake. A mechanism-based, mathematical model extended from our previous work^26^ was developed and calibrated to the live and dead cell counts to determine an inhibition constant dependent on the dose of the inhibitor. We then used the calibrated model to predict how a glucose uptake inhibitor influences tumor cell growth by limiting glucose access. Finally, the accuracy of this mechanism-based model was compared to four common machine learning models to study the relative advantages and disadvantages of the two approaches to predictive modeling.

## Materials and methods

### Cell culture and treatment with glucose uptake inhibitor Cytochalasin B

We obtained the triple negative breast cancer cell line, MDA-MB-231^33^, from the American Type Culture Collection (ATCC, Manassas, VA) and maintained them in culture according to manufacturer recommendations. We seeded the MDA-MB-231 cells in ninety-six well-plates covering multiple initial confluences (30–90%) in Dulbecco’s modified eagle medium (DMEM without glucose, sodium pyruvate, HEPES, L-glutamine and phenol red, Thermo Fisher Scientific, Waltham, MA) the day prior to image acquisition began. The cells were incubated in the IncuCyte S3 live cell imaging system (Essen BioScience, Ann Arbor, MI) at 37 °C for 4 days, supplied with 5% CO_2_ and air. Before image acquisition began, we changed the cell culture medium to DMEM with designated glucose (0.5 mM, 1 mM, 2 mM, 5 mM, and 10 mM, Sigma-Aldrich, St. Louis, MO) and Cytochalasin B (0 μM, 2 μM, or 10 μM, Sigma-Aldrich, St. Louis, MO) concentrations. (Cytochalasin B competitively inhibits the transport of glucose with high efficiency as a glucose uptake inhibitor^34^.) After the medium change, we added Cytotox Red (Essen BioScience, Ann Arbor, MI) to the medium to monitor cell death. (Cytotox Red enters the cell plasma through the disintegrated membrane of dead cells and then binds to DNA to emit fluorescent signals.) Analysis of the time-lapse fluorescent images allowed the quantification of cell death over time. We prepared four replicates for each initial condition.

With the IncuCyte S3 live cell imaging system, we collected phase-contrast and red fluorescent (excitation wavelength: 585 nm and emission wavelength: 635 nm) images of whole-wells every 3 h for 4 days. While details are provided elsewhere^26^, the salient features are as follows. A 4 × objective was used to image both channels. The whole-well images were acquired through stitching of multiple images collected by raster scanning across each well. This allows the tracking of both the total confluence (percentage of area covered by cells) and area covered by dead cells (labeled with Cytotox Red) during the entire experiment.

### Image processing for cell segmentation

We used Matlab (The Mathworks, Inc., Natick, MA) to perform cell segmentation and generate the data for our modeling, including the time-resolved confluence curves from each well for both live and dead cells. Briefly, we masked the region of interest (ROI) and converted RGB (red, green, blue) images to grayscale for images from both channels. For phase-contrast images we binarized the image based on pixel-wise signal intensity. For fluorescent images we applied a sliding block for edge detection and a Gaussian filter for area smoothing then normalized and binarized the images. Complete details are provided in our previous work^26^.

### Mechanism-based model

We begin with the most parsimonious model selected from our previous study, which used a function of glucose level (details on model selection and derivation are provided in ref. ^26^) to model the temporal change of tumor cell number and extend it to include the effect of treatment with Cytochalasin B. Our complete model is described by a series of coupled ODEs to calculate the number of live, *N*(*t*), and dead cells, *D*(*t*), at any given time point, *t*, as shown below:

1 $$\frac{dN(t)}{{dt}} = k_{p} N(t)\left( {1 - \frac{N(t)}{\theta }} \right)S_{p} \left( {G_{acs} (t)} \right) - k_{d} N(t)S_{d} \left( {G_{acs} (t)} \right) - k_{bys} N(t)\left( {\frac{D(t)}{{D(t) + N(t)}}} \right)$$

2 $$\frac{dD(t)}{{dt}} = k_{d} N(t)S_{d} \left( {G_{acs} (t)} \right) + k_{bys} N(t)\left( {\frac{D(t)}{{D(t) + N(t)}}} \right)$$

3 $$\frac{{dG_{total} (t)}}{dt} = - vN(t)\left( {\frac{{G_{acs} (t)}}{{G_{acs} (t) + G^{*} }}} \right)$$

4 $$S_{d} \left( {G_{acs} (t)} \right) = \left( {1 - \frac{{G_{acs} (t)}}{{G_{acs} (t) + G_{\min } }}} \right)\tanh (t)$$

5 $$S_{p} \left( {G_{acs} (t)} \right) = 1 - \left( {1 - \frac{{G_{acs} (t)}}{{G_{acs} (t) + G_{\min } }}} \right)\tanh (t)$$

6 $$G_{acs} (t) = G_{total} (t)\frac{1}{{1 + G_{in} N(t)}}.$$

**Figure 1:**
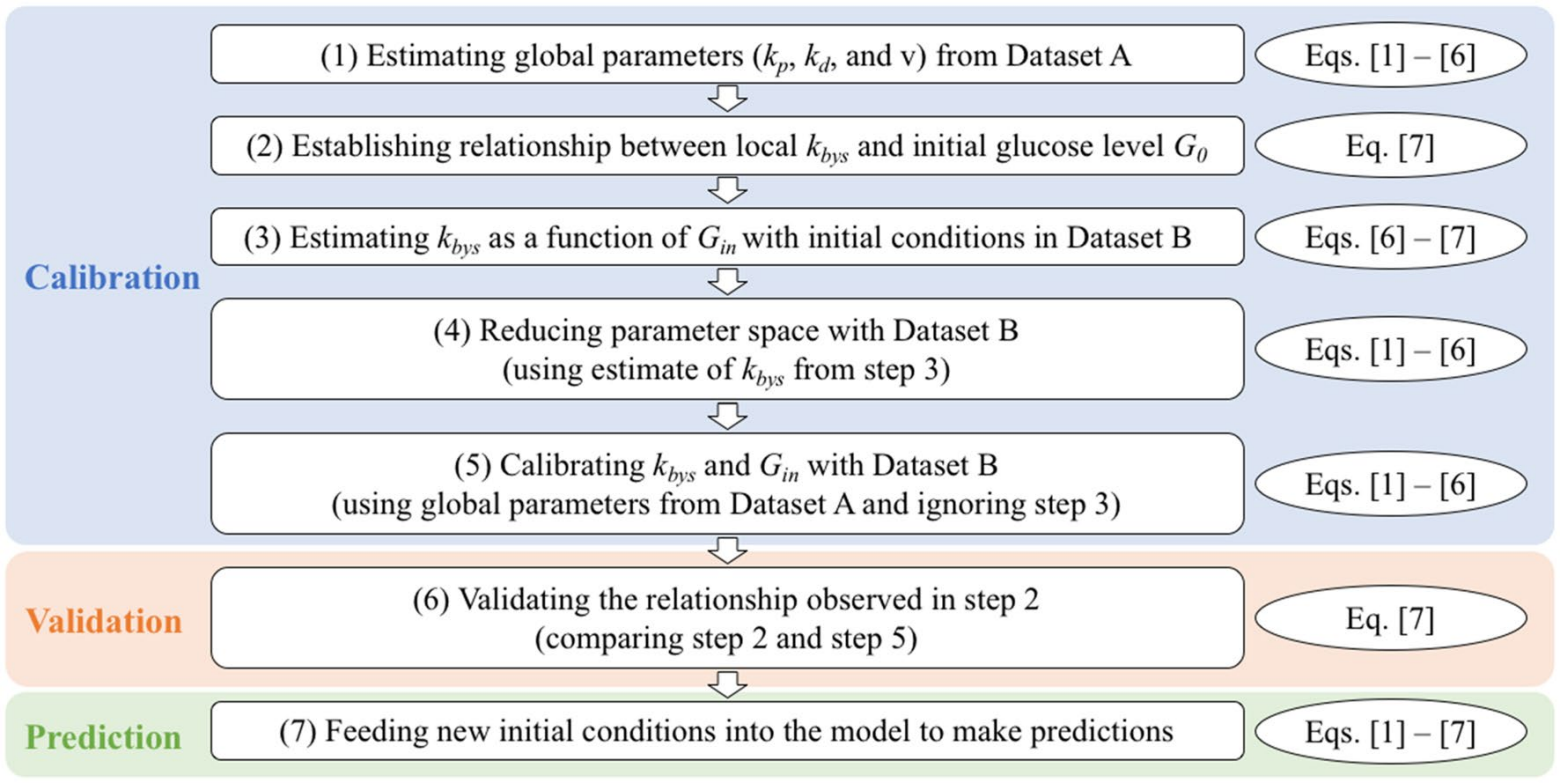
An overview of the approach used in the mechanism-based approach. The figure shows the three main steps of calibration, validation, and prediction steps. Descriptions are shown in boxes and the associated equations are shown in ellipses next to each box. These steps are referred to frequently in the Materials and Methods section.

All model functions, variables, and parameters are described in Table 1. Additionally, the reader is encouraged to consult Figs. 1 and S1 for a brief and detailed, respectively, overview of our approach.

**Table 1:** The definitions, units, source, and value for the model parameters.

Parameter	Definitions	Units	Source	Value
*k*_*p*_	Proliferation rate	day^−1^	Assigned from previous study^26^	0.14
*k*_*d*_	Death rate due to starvation	day^−1^	Assigned from previous study^26^	0.041
*k*_*bys*_	Death rate due to bystander effect	day^−1^	Calibrated	–
*θ*	Carrying capacity	Cells	Assigned from literature^33^	8 × 10^4^
*v*	General glucose consumption	mM·cell^−1^·day^−1^	Assigned from previous study^26^	4.48 × 10^–5^
G*	Michaelis–Menten constant	mM	Assigned from literature^16^	0.5
*G*_*min*_	Minimum glucose level for uptake	mM	Assigned from literature^16^	0.01
*G*_*in*_	inhibition constant	cell^−1^	Calibrated	–

As Cytochalasin B is a glucose uptake inhibitor, we introduced *G*_*acs*_(*t*) to describe the glucose level accessible to cells (i.e., the *effective* glucose concentration within the extracellular environment, due to the impact of the glucose uptake inhibitor) at time *t*, in comparison to *G*_*total*_(*t*), which is the actual glucose level in the system (i.e., the extracellular glucose concentration) at time *t*. The three terms on the right-hand side of Eq. (1) describe logistic tumor cell growth, tumor cell death induced by glucose depletion, and tumor cell death induced by the bystander effect^26,35,36^, respectively. Death can be induced in the remaining live cells with factors^35,36^ released by dead cells into the environment. We considered this phenomenon as a manifestation of the bystander effect and included it in the model^26^ as cell death was consistently underestimated when only considering glucose depletion. Equation (2) describes the accumulation of dead cells from both sources of death (i.e., glucose depletion and the bystander effect). Equation (3) describes the change of total glucose concentration due to the consumption by tumor cells as a function of accessible glucose concentration, which is the glucose level cells could actually utilize. Equations (4) and (5) are state functions, scaling the proliferation rate or glucose depletion induced death rate in real-time as a function of glucose level accessible to cells, where *G*_*min*_ is minimal glucose level required to proliferate. Equation (6) describes the relationship between the accessible glucose concentration and total glucose concentration, where *G*_*in*_ is an inhibition constant dependent on the dose of Cytochalasin B. When there is no treatment, *G*_*acs*_ is equal to *G*_*total*_ and this extended model becomes the same as the baseline model. This formulation assumes Cytochalasin B only effects the ability of tumor cells to access glucose, and does not directly inhibit glycolysis, pyruvate metabolism, or oxidative phosphorylation.

### Machine learning models

The prediction of tumor cell growth can be formulated as a supervised learning task; that is, as a regression problem given a set of input features. In this study, we considered the number of both live and dead tumor cells at time zero (i.e., *N*(*t* = 0) and *D*(*t* = 0)), initial glucose concentration (i.e., *G*_*total*_(*t* = 0)), Cytochalasin B dose, and the time of measurement as input features. The number of tumor cells at any given time point for both live and dead cells (i.e., *N*(*t*) and *D*(*t*)) are the prediction targets.

We employed four commonly used machine learning algorithms: multivariate linear regression, *k*-nearest-neighbor regression, decision tree regression, and random forest regression. Multivariate linear regression seeks to estimate the result of a variable of interest, using a number of explanatory variables^37^. It is a parametric approach as it assumes a linear functional form mapping from the input variables to the output variable(s). While parametric approaches are generally easy to fit with a small set of coefficients, they rely on a strong assumption about the form of the linear function, which may not always be correct. Therefore, we also explored non-parametric approaches that do not assume an explicit functional form, thereby allowing greater flexibility. The first non-parametric approach we introduced was the *k*-nearest-neighbor method^38,39^. This algorithm calculates a weighted average (equal to the inverse of the distance) of the *k*-nearest-neighbor as an estimate for numerical variables. While the non-parametric approaches are more flexible, they can often be difficult to understand and interpret biologically. Therefore, we included a decision tree regression approach^40^, which takes observations about a sample (represented as the branches) to estimates of the target variable (represented as the leaves). Decisions trees are more straightforward to interpret as they follow simple decision rules constructed depending on the data features. Finally, we investigated random forest regression^41,42^ which is an ensemble learning method that trains multiple decision-tree models to achieve better performance in prediction compared to results from a single model^43–45^. This algorithm employs bootstrap aggregating (or bagging^46^) to generate multiple random samples with replacement to train multiple decision trees and then uses the average prediction from each tree to yield a final prediction. Random forest regression can correct for overfitting^47–49^ resulting from a decision trees analysis^50^, but at the expense of a more difficult to interpret single decision tree.

All machine learning models were implemented in Jupyter Notebooks with the Python library Scikit-learn. To implement the models, the number of cells were first normalized to a value between 0 and 1 (i.e., confluence) by scaling to the carrying capacity (Table 1). Splitting the timeseries data into a training and validation set requires special consideration to avoid data leakage^51^ (i.e., when future information is included in the training set, but similar data is not available when the model is used for prediction, resulting in unrealistically high performance in training and potentially poor generalization on unseen data). Our data was split into a training set containing 75% of the samples and a separate validation set containing the remaining 25% (more details can be found in section "Training and validation"). Models were trained only on the training set to estimate model parameters. The trained model was then evaluated on the validation set. As our interest lies in comparing with the mechanism-based model, it is crucial to be consistent on the features we use, properties that we can measure or control in experiments. Therefore, we only used the five features described at the beginning of this section (i.e., *N*(*t* = 0), *D*(*t* = 0)), *G*_*total*_(*t* = 0), Cytochalasin B dose, and the time of measurement) and did not apply further feature selection or dimension reduction. Default hyperparameters as provided by the Scikit-learn library were used for these algorithms and no parameter tuning was performed, allowing us to evaluate their basic performance on our dataset and establish a baseline comparison with the mechanism-based model.

### Model calibrations

Throughout the following section, the reader is encouraged to refer to Figs. 1 and S1. Figure 1 provides an overview of the methodology used in the mechanism-based model, while Fig. S1 provides a more detailed schema of the whole calibration and prediction process.

We used two datasets for our model calibration. Dataset A was obtained from our previous work^26^ and consists of 120 pairs of confluence time courses for live and dead cells from each sample. In Dataset A, the cells were seeded at low, intermediate, and high initial confluences with 10 initial glucose concentrations (0, 0.1, 0.2, 0.5, 0.8, 1, 2, 5, 8, and 10 mM) and were not treated with Cytochalasin B. Dataset B was obtained from new experiments and consist of 180 pairs of confluence time courses for live and dead cells. In Dataset B, the cells were seeded at low, intermediate, and high initial confluences with five initial glucose concentrations (0.5, 1, 2, 5, and 10 mM). Of the 180 wells imaged, 60 wells were not treated, 60 wells were supplied with 2 μM Cytochalasin B, and 60 wells were supplied with 10 μM Cytochalasin B. In both datasets, there were four replicates for each initial condition. Note that for the mechanism-based model, the cell confluences (of both live and dead cells) are multiplied by the carrying capacity *θ* (i.e., the maximum number of cells that can physically fit in the area, Table 1) and converted into the number of live and dead cells (*N*(*t*) and *D*(*t*), respectively).

#### Calibration of the mechanism-based model with Dataset A

The model described in section "Mechanism-based model" (i.e., Eqs. (1)–(6)) was first calibrated to experimental data (i.e., time-resolved measurements of tumor cell number for live and dead cells) from Dataset A, using the initial glucose level and confluence as the initial conditions (Fig. S1, blue arrows, step 1). It was assumed (based on the results from previous work^26^) that the proliferation rate, *k*_*p*_, the consumption rate of glucose, *v*, and the glucose depletion induced death rate, *k*_*d*_, were characteristics of the cell line, while the bystander effect induced death rate, *k*_*bys*_, was dependent on initial glucose level. Therefore, we obtained estimates for three global parameters (i.e., a set of parameters shared and applicable across the entire dataset; *k*_*p*_, *k*_*d*_, and* v*) and a local parameter (i.e., a parameter specific for a given sample; *k*_*bys*_) for each of the 120 time courses in Dataset A. The confidence intervals for all three parameters were also computed.

#### Establishing relationships between mechanism-based model parameters and initial glucose level

The estimates of *k*_*bys*_ obtained from section "Calibration of the mechanism-based model with Dataset A" were fit to Eq. (7):

7 $$k_{bys} = k_{bys,0} \exp ( - \alpha G_{0} ) + \beta ,$$

where *k*_*bys,0*_ is the maximum *k*_*bys*_ rate when there is sufficient glucose, *α* represents the dependence on the initial glucose level, *G*_*0*_ represents the initial glucose level, and *β* represents a baseline offset (Fig. S1, orange arrows, step 2). This follows the approach developed in our previous work^26^, assuming *k*_*bys*_ presents an exponential decay depending on the initial glucose concentration. With the estimates of these three parameters, Eq. (7) can be used to estimate *k*_*bys*_ for a new initial condition (i.e., Dataset B) not contained in the training dataset (i.e., Dataset A). With new initial conditions from Dataset B, Eq. (6) was used to represent the initial accessible glucose level *G*_*acs,0*_, and it is substituted for *G*_*0*_ in Eq. (7). *k*_*bys*_ is therefore represented as a function of *G*_*in*_ for each sample in Dataset B (Fig. S1, green arrows, step 3).

#### Calibration of the mechanism-based model with Dataset B to reduce the parameter space for ***G***_***in***_

The model described in section "Mechanism-based model" was calibrated to experimental data (180 pairs of time-resolved measurements of tumor cell number for live and dead cells) from Dataset B, using the initial glucose level and confluence as the initial conditions (Fig. S1, red arrows, step 4). In this calibration, the three global parameters *k*_*p*_, *k*_*d*_, and* v*, were replaced with the estimates from section "Calibration of the mechanism-based model with Dataset A", while the local parameter *k*_*bys*_ was obtained from step 3 as described in section "Establishing relationships between mechanism-based model parameters and initial glucose level". In our modeling, we have the ability to model *G*_*in*_ as a global parameter (i.e., one value for all samples included in the experiments) or a local parameter (i.e., one value for each sample), or somewhere in between those two extremes. Through our simulation experiments, we found having two values, one for each Cytochalasin B concentration, yielded the best model performance. With this approach we can avoid having too much flexibility in the model and risk overfitting, but also make sure the model is capable of capturing the trends apparent in the data. Therefore, in step 3 we only estimate the inhibition constant *G*_*in*_ as a global parameter, assuming *k*_*bys*_ still follows the patterns observed in our previous study^26^; i.e., *k*_*bys*_ decreases with increasing initial glucose level following an exponential decay. We obtained an estimate of *G*_*in*_ for cells treated with 2 μM and 10 μM of Cytochalasin B, respectively, assuming the inhibition is dose dependent. In this step *k*_*bys*_ was considered as a known parameter (fixed for each sample, assigned with values from Eq. (7)), instead of a free parameter that needs calibration, to reduce the model flexibility and therefore the parameter space of *G*_*in*_ (i.e., to narrow down the potential values of *G*_*in*_). Confidence intervals of *G*_*in*_ were also obtained.

#### Calibration of the mechanism-based model with Dataset B to estimate ***k***_***bys***_ and ***G***_***in***_

The model described in section "Mechanism-based model" was calibrated to experimental data (180 pairs of time-resolved curves of tumor cell number for live and dead cells) from Dataset B, using the initial glucose level and confluence as the initial conditions (Fig. S1, purple arrows, step 5). The three global parameters *k*_*p*_, *k*_*d*_, and* v*, were replaced with their estimates from section "Calibration of the mechanism-based model with Dataset A". However, as opposed to the approach described in section "Calibration of the mechanism-based model with Dataset B to reduce the parameter space for Gin", *k*_*bys*_ was treated as a free local parameter and estimated together with the global parameter *G*_*in*_, with the confidence intervals obtained from section "Calibration of the mechanism-based model with Dataset B to reduce the parameter space for Gin" serving as the bounds for estimating *G*_*in*_.

#### Validation of the relation between local ***k***_***bys***_ and accessible glucose concentrations

The estimates of *G*_*in*_ obtained from the last calibration performed according to section "Calibration of the mechanism-based model with Dataset B to estimate kbys and Gin", along with the initial total glucose level *G*_*total*_(*t* = 0) and number of live cells *N*(*t* = 0) were used to calculate the initial accessible glucose level *G*_*acs*_(*t* = 0) using Eq. (6). The estimates of *k*_*bys*_ as a local free parameter from the same calibration were compared to both *G*_*total*_(*t* = 0) and *G*_*acs*_(*t* = 0) to determine if they follow an exponential decay as we discovered in our previous work^26^. If the pattern is again observed, then the estimates of *k*_*bys*_ were fit to Eq. (7) to update the estimates of *k*_*bys,0*_, *α*, and *β* (Fig. S1, brown arrows, step 6).

#### Evaluation of the performance of the mechanism-based model

We introduced five criteria, namely the coefficient of determination (*R*^2^), mean percent error over the time course, percent error at the end of experiment, mean error over the time course, error at the end of experiment (more details on these errors are described in Table S1) to evaluate model performance and compare across different approaches.

#### Prediction with the mechanism-based model

Given new initial conditions of glucose level, confluence, and dose of Cytochalasin B, we can predict tumor cells with the calibrated model (Fig. S1, cyan arrows, step 7). The initial accessible glucose level *G*_*acs*_(*t* = 0) can be calculated using Eq. (6) and then used in Eq. (7) to calculate *k*_*bys*_. With the initial confluence and initial accessible glucose level, we can solve Eqs. (1)–(6) to run the model forward and obtain time-resolved curves of live and dead tumor cells.

#### Validation of the accessible glucose level

We sought to validate the assumption that the glucose level accessible to the tumor cells was lower than the actual glucose level in the system, and then provide an estimate of the accessible glucose level for the cells treated with Cytochalasin B. To perform this test, we compared the confluence time courses of the tumor cells with different initial glucose levels but with no exposure to Cytochalasin B. The concordance correlation coefficient (CCC) was employed as a measurement of the agreement between the two sets of time-resolved curves. We hypothesized that cells growing in environments with similar glucose levels would present similar growth curves. Therefore, we compared time-resolved cell number curves of tumor cells with treatment to time-resolved cell number curves of tumor cells without treatment and with similar initial confluences, but with different initial glucose levels. The growth curve without treatment that yielded the highest CCC when compared to the growth curve with treatment provided the best estimate of the accessible glucose level for the treated cells.

### Training and validation

#### Training and test sets

The data set was randomly split into training (75%; *n* = 225) and validation sets (25%; *n* = 75). The training set was used to develop the predictive models and the testing set was used as an independent data set for model validation. The splitting for Datasets A and B was performed separately. The training set *A*_*train*_ (75%; *n* = 90) and the validation set *A*_*valid*_ (25%; *n* = 30) were divided from Dataset A, while the training set *B*_*train*_ (75%; *n* = 135) and the validation set *B*_*valid*_ (25%; *n* = 45) were divided from Dataset B.

#### Training and validation for the mechanism-based model

The model described in section "Mechanism-based model" (Eqs. (1)–(6)) was calibrated to *A*_*train*_ to obtain estimates for the global parameters *k*_*p*_, *k*_*d*_, and *v,* as well as estimates for the local parameter *k*_*bys*_ via the approach described in section "Calibration of the mechanism-based model with Dataset A". The estimates of *k*_*bys*_ were fit to Eq. (7) with the associated initial glucose concentration from *A*_*train*_ via the approach described in section "Establishing relationships between mechanism-based model parameters and initial glucose level" to yield estimates of *k*_*bys,0*_, *α*, and *β*. Equations (1)–(6) were first calibrated to *B*_*train*_ with the approach described in section "Calibration of the mechanism-based model with Dataset B to reduce the parameter space for Gin" to obtain a preliminary estimate of *G*_*in*_ with its confidence interval. Equations (1)–(6) were calibrated to *B*_*train*_ again with the approach described in section "Calibration of the mechanism-based model with Dataset B to estimate kbys and Gin" to obtain estimates of both *G*_*in*_, as a global parameter, and *k*_*bys*_, as a local parameter, with the confidence interval obtained from first calibration serving as the bounds for estimating *G*_*in*_. The estimates of *k*_*bys*_ were fit to Eq. (7) to estimate *k*_*bys,0*_, *α*, and *β* with the approach described in section "Validation of the relation between local kbys and accessible glucose concentrations". The initial conditions of each sample from *A*_*valid*_ and *B*_*valid*_ were used in the forward model to predict the tumor cell growth over time with the approach described in section "Prediction with the mechanism-based model" and compared with the experimental measurements in dataset *A*_*valid*_ and *B*_*valid*_. The training and validation were repeated 50 times following the steps described above; a new random train-test split was performed each time to generate the data sets needed. Each time, we calculated the *R*^2^ and errors listed in section "Evaluation of the performance of the mechanism-based model" for model performance evaluation. We report the average model performance over the 50 rounds of training and validation.

#### Training and validation for machine learning models

The training and validation process were repeated 50 times. In each round, *A*_*train*_ and *B*_*train*_ (generated as described in section "Training and test sets") were combined as the training set for the machine learning models. Similarly, *A*_*valid*_ and *B*_*valid*_ (generated as described in section "Training and validation for the mechanism-based model") were combined as the validation set for the machine learning models. In each round, each of the four machine learning models were trained on the combined training set. Predictions were obtained using the initial conditions in the combined validation set, after the machine learning models were trained. The predictions from the initial conditions given in the validation set were compared with the observations in the validation set. In each round, we calculated the coefficient of determination (*R*^2^) to for model performance evaluation. We report the average model performance (with 95% confidence interval) over 50 rounds of training and validation.

#### Learning curves for the machine learning models

For each round of training and validation, the impact from the size of the training set was studied. The four machine learning models were trained on training sets with size increasing from 5 to 100% (with a step size of 5%) of the complete training set (randomly selected) and the model performance on both training and validation sets were evaluated. This process was repeated for 50 rounds.

### Statistical analysis

Tumor cell growth time courses were obtained from four experiments for each set of initial conditions, and each point in each time course consisted of a mean ± 95% confidence interval (a one-sample Kolmogorov–Smirnov test confirmed normality). Two‐way ANOVA was used to compare the average number of live cells for each experiment at the end of day 4 between the groups with different initial conditions. A Student's *t*-test was used to test for the statistical difference of estimates for *G*_*in*_ between the two Cytochalasin B concentrations. One‐way ANOVA was used to compare the performance (*R*^2^) of all five models. The explicit error metrics used to evaluate the mechanism-based model performance can be found in Table S1. The CCC was used to measure the agreement between two time-resolved curves. Bonferroni correction was applied to control the family-wise error rate (“FWER”) when comparing the performance between any two models, where each hypothesis is tested at a significance level of the selected alpha divided by the number of hypothesis tests to guarantee that the probability of having one or more false positives is less than alpha.

## Results

### Observations of tumor cell growth from time-resolved microscopy

**Figure 2:**
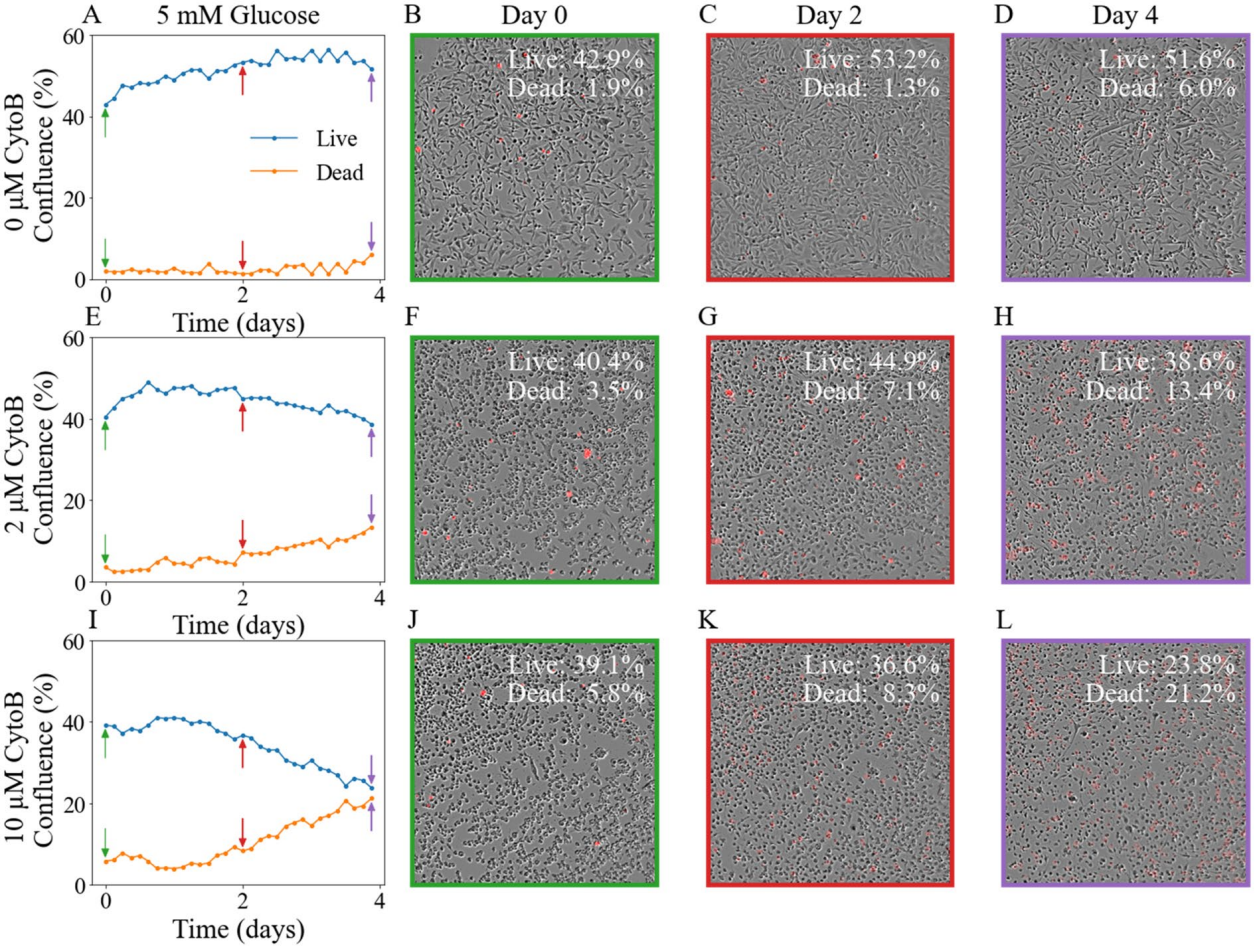
Example images and confluence time courses of cells with and without treatment with Cytochalasin B. Panels **A**, **E**, and **I** present three example confluence time courses of live (blue line) and dead (orange) cells supplied with 5 mM glucose without treatment, treatment with 2 μM of Cytochalasin B, and 10 μM of Cytochalasin B, respectively. The arrows indicate days 0, 2, and 4. The second (panels **B**, **F**, and **J**), third (panels **C**, **G**, and **K**), and last columns (panels **D**, **H**, and **L**) show the merged images of phase-contrast image and fluorescent image of cells on days 0, 2 and 4, respectively, with dead cells labeled by the Cytotox Red. In each row, the images of cell growth correspond to the confluence time course in the first column; the color of the arrows and frames match the three time points selected. The confluence of live cells and dead cells are labeled in each image.

We present representative time-resolved confluence curves of live and dead cells (Fig. 2A,E,I), along with a set of time-resolved images showing tumor cell growth and death on day 0 (Fig. 2B,F,J), day 2 (Fig. 2C,G,K), and day 4 (Fig. 2D,H,L). The cells were untreated (Fig. 2A–D), treated with 2 μM Cytochalasin B (Fig. 2E–H), or treated with 10 μM Cytochalasin B (Fig. 2I–L). Notice that the tumor cells in the first row (untreated) expanded with minimum cell death (the confluence of dead cells increased by 4.1%) over 4 days, while cells treated with Cytochalasin B (the second and third rows) expanded more slowly with more cell death (the confluence of dead cell increased by 9.9% and 15.4% for 2 μM and 10 μM of Cytochalasin B, respectively). Maximum cell death was observed for the highest dose. Observe how, in the third row, the confluence of dead cells at the end of experiment was 21.2% with the highest dose compared to 6.0% with no treatment and 13.4% with a lower dose.

### Tumor cell growth with different initial conditions and treatment

**Figure 3:**
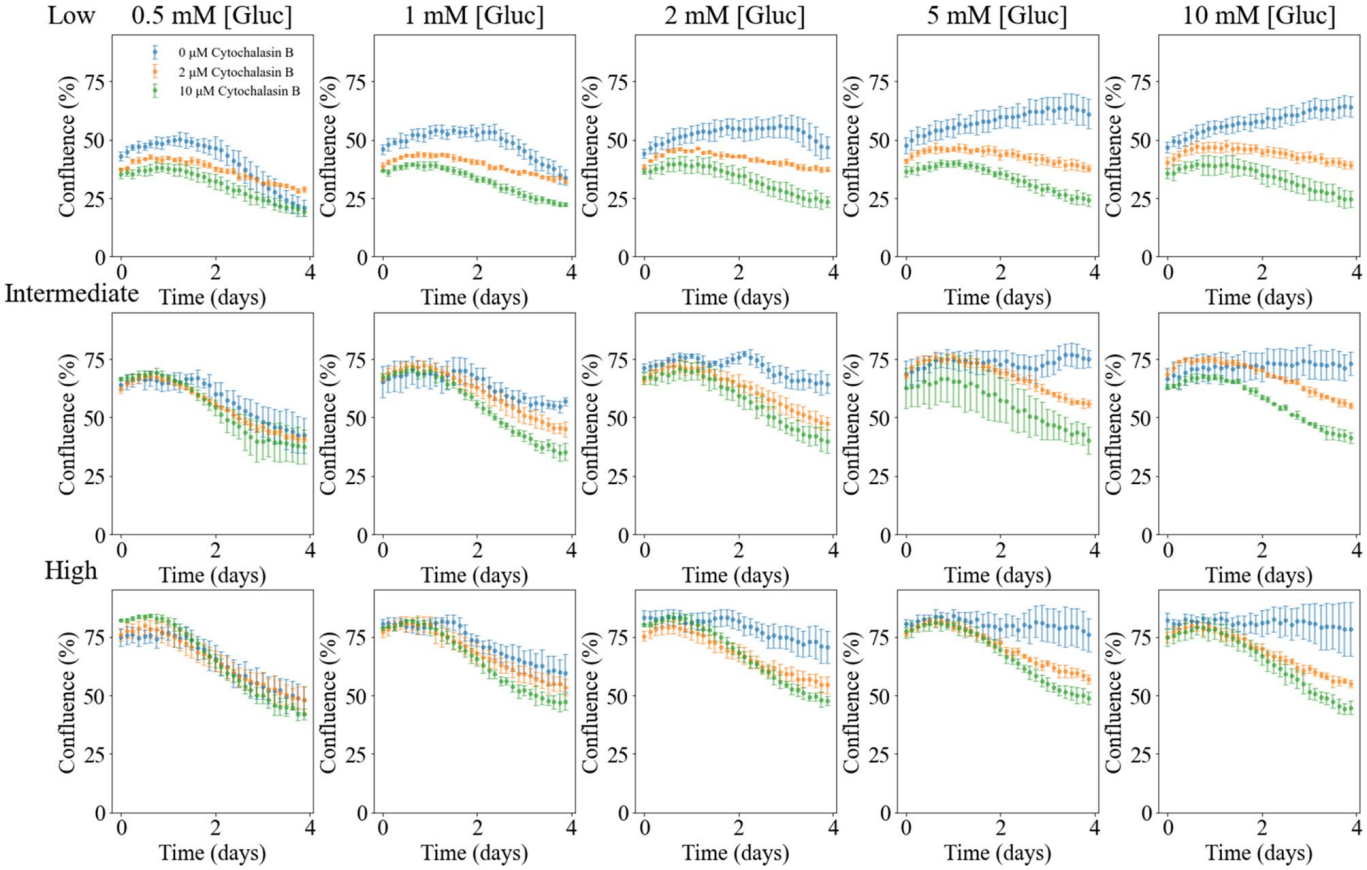
Time courses of tumor cell confluence in media with varying initial conditions. In each row, cells were seeded at the same initial confluence (top row = low, middle row = intermediate, or bottom row = high). In each column, cells were supplied with the same initial glucose concentration (0.5, 1, 2, 5, or 10 mM). In each panel, cells share the same initial confluence and glucose level while each color represents different treatments (blue: no treatment, orange: 2 μM Cytochalasin B, and green: 10 μM Cytochalasin B). These time courses provide quantitative and dynamic data on the effects of glucose accessibility (determined by the glucose level and the dose of Cytochalasin B) on tumor cell growth. The effect of Cytochalasin B is quantified by comparing the average percentage change in the number of live cells from day 0 to day 4.

Figure 3 displays time courses of the MDA-MB-231 cells with different initial confluences (i.e., seeding density) and glucose levels (0.5, 1, 2, 5, and 10 mM). Samples with low initial confluence (Fig. 3 first row), intermediate initial confluence (Fig. 3 second row), and high initial confluence (Fig. 3 third row), are shown in separate rows. In each panel, the time courses of cells not treated with Cytochalasin B (blue), treated with 2 μM of Cytochalasin B (orange), and treated with 10 μM of Cytochalasin B (green) are plotted. For each initial condition, the change (mean ± 95% confidence interval) on the number of live cells (as percentage) from day 0 to day 4 are shown in Table 2.

**Table 2:** Average percent change of the number of live cells from day 0 to day 4.

Dose (μM)	Initial confluence (%)		Initial glucose concentration (mM)				
			0.5	1	2	5	10
0	Low	45.5 ± 1.2	− 52.0 ± 6.7	− 26.2 ± 4.4	5.9 ± 6.2	28.3 ± 5.8	36.7 ± 4.7
	Intermediate	67.1 ± 0.2	− 34.1 ± 9.9	− 12.1 ± 9.2	− 9.6 ± 4.3	10.2 ± 4.3	9.5 ± 1.7
	High	80.3 ± 1.7	− 36.0 ± 5.2	− 26.3 ± 9.7	− 15.4 ± 6.7	− 6.1 ± 7.0	− 4.7 ± 11.1
2	Low	39.2 ± 0.8	− 23.2 ± 2.0	− 16.1 ± 2.5	− 3.2 ± 0.8	− 7.6 ± 2.5	− 2.1 ± 4.3
	Intermediate	66.3 ± 1.2	− 34.3 ± 3.2	− 32.5 ± 4.4	− 28.2 ± 2.4	− 18.2 ± 1.8	− 19.4 ± 2.2
	High	75.9 ± 0.9	− 36.6 ± 5.9	− 30.6 ± 3.6	− 27.8 ± 3.7	− 25.6 ± 1.8	− 26.5 ± 2.7
10	Low	36.2 ± 0.8	− 45.3 ± 3.5	− 39.2 ± 1.8	− 35.4 ± 4.9	− 33.9 ± 5.2	− 31.0 ± 6.9
	Intermediate	65.3 ± 1.9	− 43.8 ± 10.5	− 47.8 ± 5.4	− 40.4 ± 7.7	− 35.4 ± 5.1	− 34.6 ± 2.7
	High	78.6 ± 1.4	− 48.9 ± 3.0	− 39.8 ± 4.5	− 40.3 ± 2.7	− 37.1 ± 2.3	− 40.1 ± 3.9

The results in all tables are shown as mean ± 95% confidence interval.

Cells that did not receive treatment displayed a negative percentage change in the number of live cells for high initial confluence (80.3 ± 1.7%) or low initial glucose concentration (0.5 mM and 1 mM) at the end of day 4. Conversely, positive percentage changes in the number of live cells were observed for cells with low initial confluence (45.5 ± 1.2%) and higher initial glucose concentration (2 mM or higher), or cells with intermediate initial confluence (67.1 ± 0.2%) and higher initial glucose concentration (5 mM or higher). Cells treated with Cytochalasin B, regardless of the initial glucose concentration or initial confluence, also showed a negative percentage change in the number of live cells. For cells with the same initial confluence and initial glucose concentration, those treated with a higher dose (10 µM) displayed a higher percentage decrease in the number of live cells, in comparison to cells treated with a lower dose (2 µM). For cells with the same initial confluence, the average number of live cells at the end of experiment was significantly different between the groups with different initial glucose level or treatment conditions (p < 1e − 4, two-way ANOVA).

### Model calibrations

#### Estimates of inhibition constant for Cytochalasin B

By fitting the experimental data (time-resolved curves of tumor cell number for both live and dead cells) to the model (i.e., Eqs. (1)–(6)) as described in section "Calibration of the mechanism-based model with Dataset B to estimate kbys and Gin", we estimated the inhibition constant *G*_*in*_ of Cytochalasin B at 3.02 × 10^–4^ cell^−1^ for 2 μM Cytochalasin B, and 4.55 × 10^–4^ cell^−1^ for 10 μM Cytochalasin B, respectively. The inhibition constant for 2 μM Cytochalasin B was significantly lower than that for 10 μM Cytochalasin B (p < 0.05, Student’s *t*-test).

#### Local ***k***_***bys***_ trends versus accessible glucose concentrations

**Figure 4:**
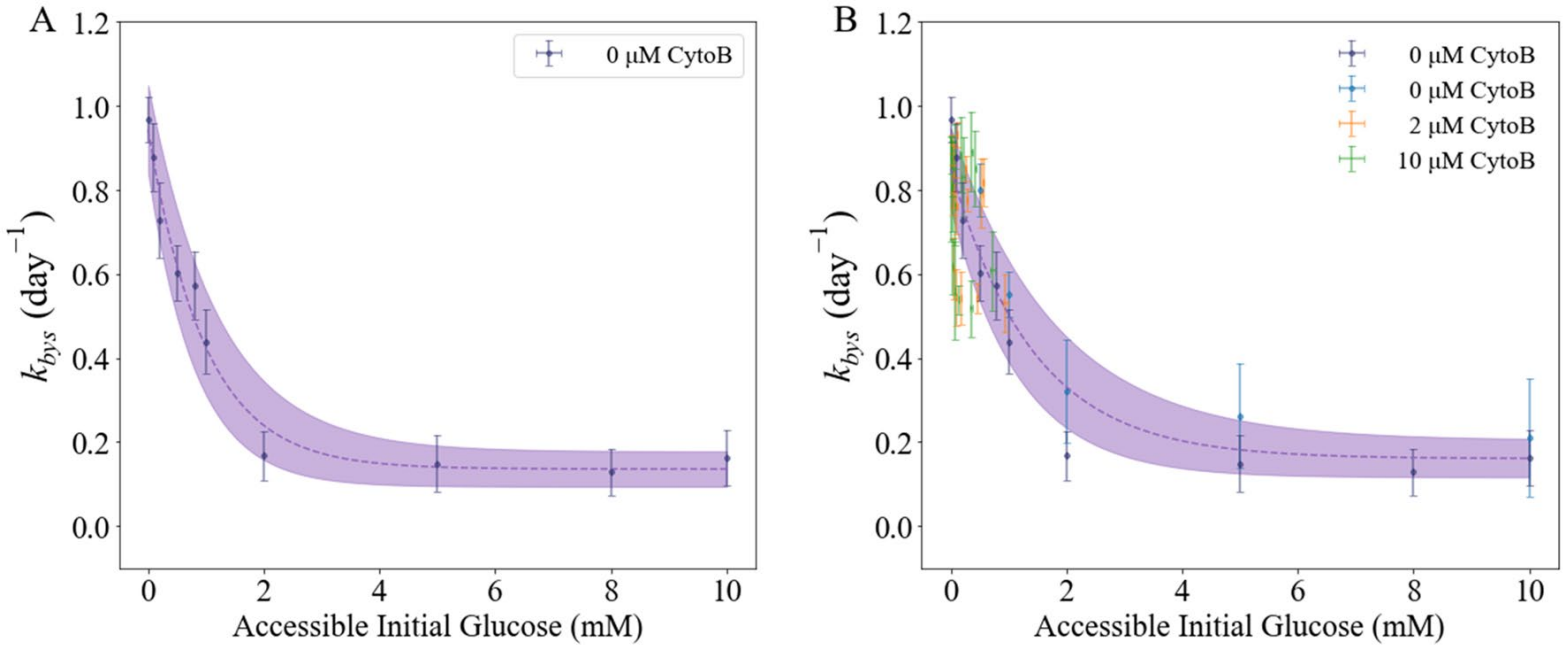
Relationship between bystander effect death rate (*k*_*bys*_) and accessible initial glucose concentrations. Panel **A** presents our previous finding^1^ on how *k*_*bys*_ decreases with initial glucose level for the MDA-MB-231 line, with shaded area between solid purple curves showing the 95% confidence interval. The dark blue dots with error bars represent the average of estimates for *k*_*bys*_ with 95% confidence interval, calibrated with the baseline model using Dataset A. Panel **B** present how *k*_*bys*_ decreases with initial accessible glucose level for the MDA-MB-231 line, with shaded area between solid purple curves showing the 95% confidence interval. The dark blue dots with error bars represent the average of estimates for *k*_*bys*_ with 95% confidence interval, calibrated with the extended model (i.e., Eqs. (1)–(7)) using Dataset A. The light blue (no treatment), orange (treated with 2 μM Cytochalasin B), and green (treated with 10 μM Cytochalasin B) dots with error bars represent the average of estimates for *k*_*bys*_ (with 95% confidence intervals), calibrated with the extended model using Dataset B. The same pattern of *k*_*bys*_ decreasing with increasing initial (accessible) glucose level was identified. The fitted curve in panels **A** and **B**, respectively, is used to assign *k*_*bys*_ as a function of initial confluence and (accessible) glucose concentration.

As described in section "Calibration of the mechanism-based model with Dataset B to estimate kbys and Gin", the estimates of *k*_*bys*_ as a free local parameter were obtained by fitting the experimental data (time-resolved curves of tumor cell number for both live and dead cells) to the model (i.e., Eqs. (1)–(6)). With the estimates of the inhibition constant *G*_*in*_ reported in the previous section, the accessible initial glucose concentrations were calculated using Eq. (6) given the initial confluence and initial total glucose level. The death rate due to the bystander effect, *k*_*bys*_, was found to decrease as the total glucose level increased with a Pearson partial inverse correlation of − 0.41 (p < 1e−4). When we consider the accessible glucose level, *k*_*bys*_ was found to decrease as the accessible initial glucose level increased (Fig. 4B), with a partial correlation coefficient of − 0.73 (p < 1e − 4). We also confirmed the relation between *k*_*bys*_ and the accessible initial glucose level discovered in previous work^26^ (Fig. 4A).

#### Fitting quality of the extended model

**Figure 5:**
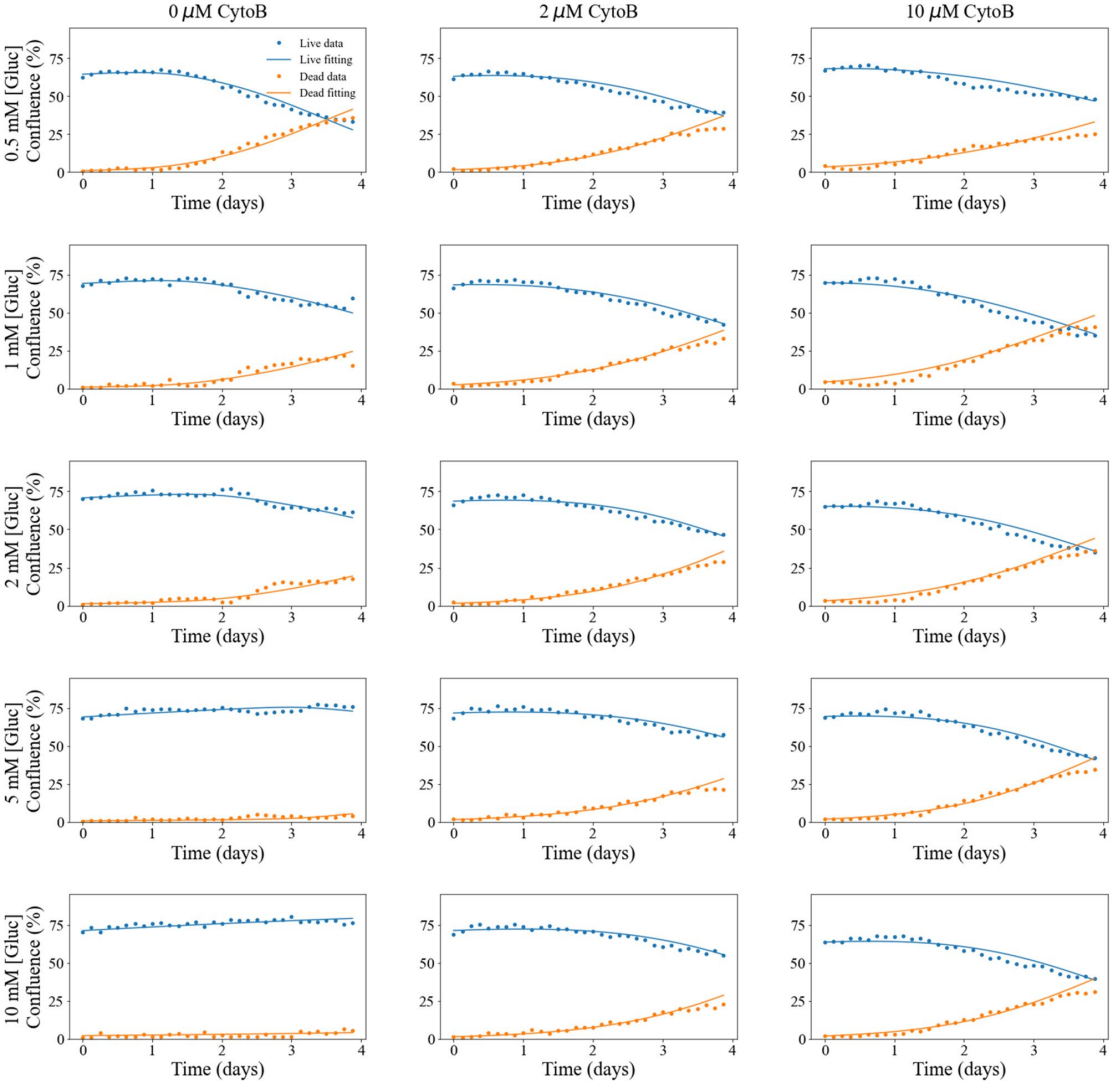
Model fitting for MDA-MB-231 cells with different glucose levels and treatments. In each row, cells were supplied with the same initial glucose concentration (0.5, 1, 2, 5, or 10 mM, respectively). In each column, the same treatment options were applied to the cells (no treatment, 2 μM Cytochalasin B, or 10 μM Cytochalasin B, respectively). In each panel, measured data are shown in dots with curves from the model shown as curves. The results for live cells and dead cells are shown in blue and orange, respectively. Our model is capable of capturing the different growth curves from different initial conditions with different treatments.

As described in section "Model calibrations", Eqs. (1) – (6) were fit to the experimental data. The errors listed in section "Evaluation of the performance of the mechanism-based model" are reported in Table 3. The model was able to provide an accurate description of the time-resolved curves of live and dead tumor cell number across various initial conditions covering a wide range of glucose levels and seeding densities. Both the mean percent error over the entire experiment and the percent error at the end of experiment, were < 1% for both live and dead cells. Both the mean error over the entire experiment and error at the end of experiment, were < 0.3% for both live and dead cells. Example results of model fittings to the measured data are shown in Fig. 5.

**Table 3:** Summary of fitting quality with the extended model (Eqs. (1)–(7)).

	Live	Dead
Mean percent error	0.25 ± 0.52	− 0.38 ± 0.56
Percent error at end of experiment	0.90 ± 1.08	− 0.65 ± 0.73
Mean error	0.09 ± 0.12	− 0.08 ± 0.10
Error at end of experiment	0.25 ± 0.14	− 0.20 ± 0.13

#### Accessible glucose level

**Figure 6:**
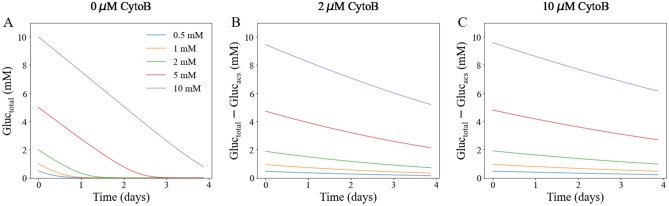
Comparison of total and accessible glucose levels from model calibration. Panel **A** presents the change of glucose level over time for cells without treatment. In this case, the total glucose level is the same as the accessible glucose level. Panels **B** and **C** present the differences between the total and accessible glucose levels for cells treated with 2 μM and 10 μM Cytochalasin B, respectively. In each panel, the same treatment options were applied, and cells supplied with different initial glucose levels (0.5, 1, 2, 5, and 10 mM) are shown in different colors. The differences decreased over time as the cells consume glucose. For cells treated with a higher dose, the differences decreased at a lower rate.

With the model calibrated, we were able to calculate the total glucose level in the system as well as the glucose level accessible to the tumor cells. The accessible glucose levels were reduced compared to the total glucose levels, depending on the administered dose of Cytochalasin B. We compared the difference between the total glucose level and the accessible glucose level over time (Fig. 6). For cells with the same initial glucose level, the differences were always higher in cells treated with the 10 µM of Cytochalasin B compared to cells treated with 2 µM of Cytochalasin B. For cells treated with the same dose, the differences were always higher for cells with higher initial glucose level. The differences decreased over time as the cells consume glucose. For cells treated with the higher dose, the differences decreased at a lower rate. For example, the difference for cells with 10 mM initial glucose and treated with 10 µM Cytochalasin B decreased from 9.6 to 6.2 mM, while cells with 10 mM initial glucose and treated with 2 µM Cytochalasin B decreased from 9.5 to 5.2 mM.

**Figure 7:**
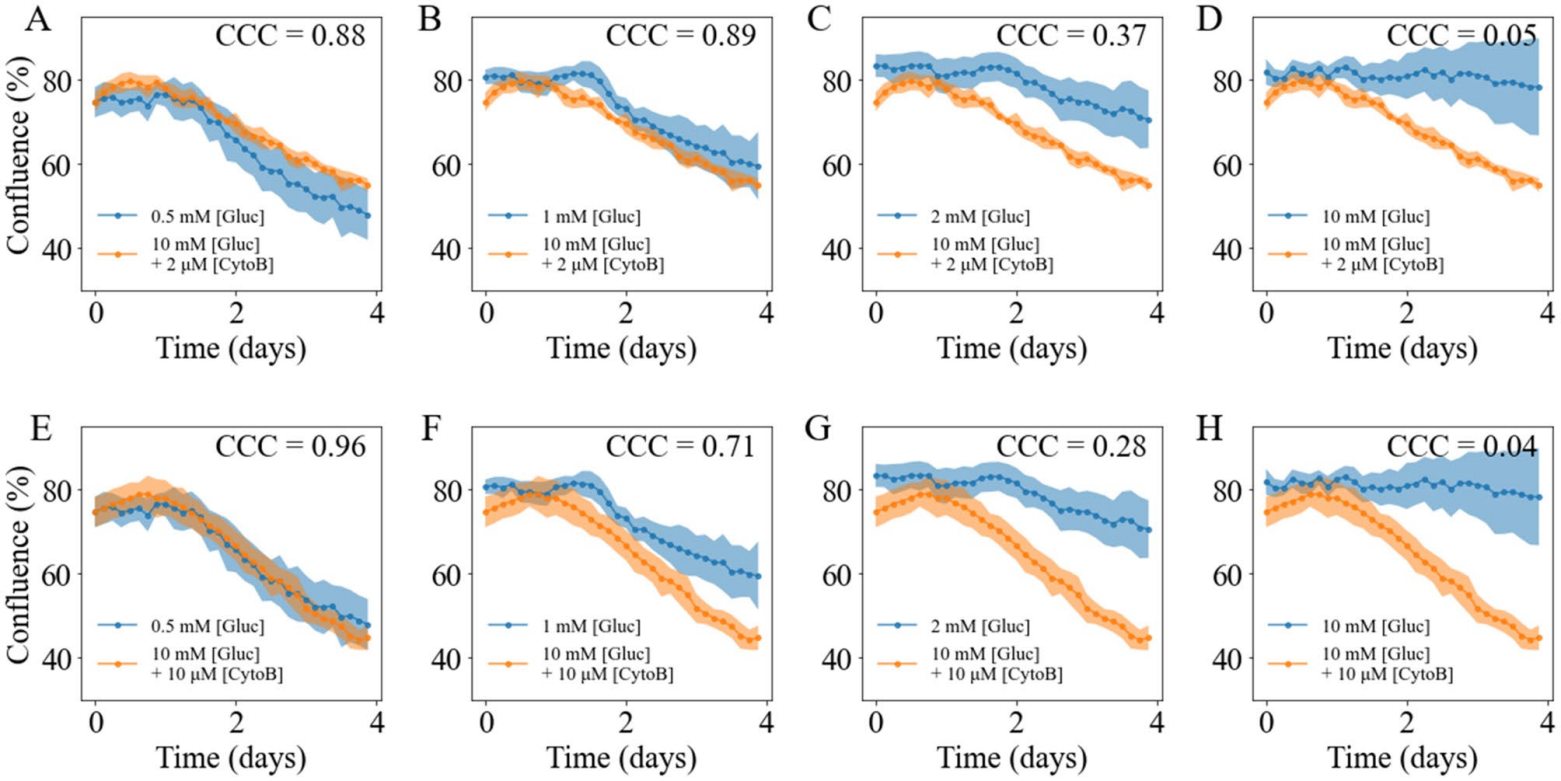
Comparison of confluence time courses between treated and untreated groups with different glucose levels. Panels **A**–**D** present the comparison of confluence time courses supplied with 10 mM glucose and treated with 2 μM Cytochalasin B, and cells supplied with different glucose levels (**A** 0.5 mM, **B** 1 mM, **C** 2 mM, and **D** 10 mM) and without treatment. Panels **E**–**H** present the comparison of confluence time courses of cells supplied with 10 mM glucose and treated with 10 μM Cytochalasin B and cells supplied with different glucose levels (**E** 0.5 mM, **F** 1 mM, **G** 2 mM, and **H** 10 mM) without treatment. In each panel, confluence time course of cells treated are shown in orange while confluence time course of cells without treatment are shown in blue. The more curves are aligned with each other, the higher their CCC. A high CCC between a treated and an untreated group suggests that the accessible glucose level in the treatment group is close to the glucose level in the untreated group.

Without a direct measurement of the accessible glucose level, we compared the confluence time courses of cells supplied with a high glucose level but treated with Cytochalasin B to cells supplied with different glucose levels but not treated with Cytochalasin B (Fig. 7). The reported CCCs quantify the similarity between two confluence time courses. The confluence time courses of cells initially supplied with 10 mM glucose and treated with 2 μM Cytochalasin B exhibit the highest CCC with cells initially suppled with 0.5 mM glucose (CCC = 0.88) and cells supplied with 1 mM glucose (CCC = 0.89). Conversely, cells supplied with 2 mM and 10 mM glucose had much lower CCC values (0.37 and 0.05, respectively). This suggests the accessible glucose level for cells supplied with 10 mM glucose and treated with 2 μM Cytochalasin B was close to 1 mM. The confluence time course of cells initially supplied with 10 mM glucose and treated with 10 μM Cytochalasin B presents the highest CCC with cells suppled with 0.5 mM glucose (CCC = 0.96). Conversely, cells initially supplied with 1 mM, 2 mM, and 10 mM glucose present lower CCC values (0.71, 0.28, and 0.04, respectively) when compared to cells initially supplied with 0.5 mM glucose. This suggested the accessible glucose level for cells supplied with 10 mM glucose and treated with 10 μM Cytochalasin B was close to 0.5 mM. Using this approach, we confirmed that the accessible glucose level for cells treated with the 10 μM of Cytochalasin B was lower than cells treated with 2 μM of Cytochalasin B.

### Evaluation of the performance of the mechanism-based model through training and validation

Table 4 summarizes the model performance in the training stage. The average mean percent error over the entire experiment span, and the average percent error at the end of experiment for live cells were both < 3%. Although the average mean percent error over the entire experiment span, and the percent error at the end of experiment for dead cells were over 18%, the average mean errors over the entire experiment span and average error at the end of experiment for both live and dead cells were < 3.5%. This suggests the higher percent error of dead cells can be explained by the small number of dead cells in comparison to the number of live cells; for example, an error of 1% in the confluence of dead cells would result in a 20% percent error for a sample with confluence of dead cells at 5%.

**Table 4:** Summary of model calibration across 50 training sets.

	Live	Dead
Mean percent error	1.18 ± 0.15	18.13 ± 0.35
Percent error at end of experiment	2.78 ± 0.24	19.10 ± 1.05
Mean error	0.28 ± 0.08	0.83 ± 0.03
Error at end of experiment	0.31 ± 0.09	3.28 ± 0.10

Table 5 reports the model performance in the validation stage. The average mean percent error over the entire experiment, and the average percent error at the end of experiment for live cells were both < 2.5%. Although the average mean percent error over the entire experiment, and the percent error at the end of experiment for dead cells were over 19%, the average mean error over the entire experiment and average error at the end of experiment for both live and dead cells were < 4.5% for both live and dead cells. Again, this indicates that the higher percent error of dead cells can be explained by the small number of dead cells in comparison to the number of live cells.

**Table 5:** Summary of prediction error across 50 rounds of training and validation.

	Live	Dead
Mean percent error	0.83 ± 0.22	19.81 ± 0.76
Percent error at end of experiment	2.00 ± 0.38	25.26 ± 2.04
Mean error	0.08 ± 0.10	0.81 ± 0.07
Error at end of experiment	− 0.20 ± 0.12	4.15 ± 0.18

### Comparison of model performance between mechanism-based and machine learning models

**Figure 8:**
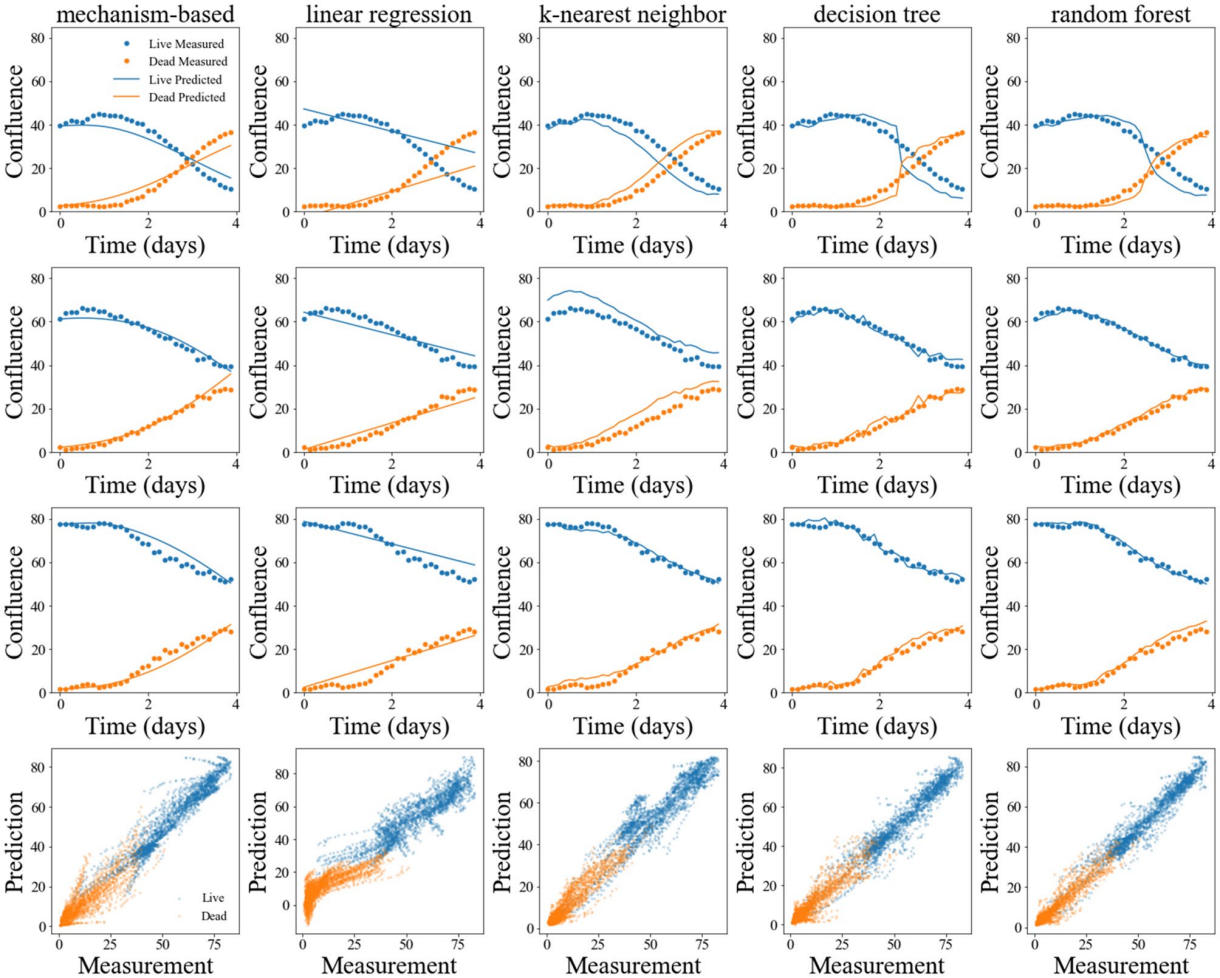
Model prediction with the mechanism-based and machine learning models. In each column, the results from the same model are shown. In the first three rows, examples of the comparison between the experimental measurement and predicted confluence time course from each model are shown. In each of these three rows, measurement from the same well is used. Measurement from three wells with low (first row), intermediate (second row), and high (third row) initial confluence were used as examples. In each panel, measured data are shown with dots, with live cells shown in blue and dead cells shown in orange; model predictions are shown as curves, with lives cells shown in blue and dead cells shown in orange. The bottom row shows scatter plots of prediction against measurement for every well from each of the five models, with results for live cells shown blue and dead cells shown in orange. The random forest model yielded the best performance in predicting tumor cell growth.

The coefficient of determination (*R*^2^) was used to compare the performance across the mechanism-based model and four machine learning models. The results are shown in Table 6. The model performance for predicting live cells alone, dead cells alone, or both live and dead cells were compared. The *R*^2^ value of the mechanism-based model calibrated on the training data set was 0.9627 ± 0.0000, while the values for the decision tree model, random forest model, *k*-nearest-neighbor regression model, and the linear regression model were 1.0000 ± 0.0000, 0.9991 ± 0.0000, 0.9283 ± 0.0014, and 0.7027 ± 0.0019, respectively. The random forest model performed the best at predicting tumor cell growth on the validation set with an *R*^2^ of 0.9260 ± 0.0028, followed by the decision tree, *k*-nearest-neighbor regression, mechanism-based, and linear regression models with *R*^2^ values of 0.8892 ± 0.0046, 0.8408 ± 0.0075, 0.7696 ± 0.0130, and 0.6903 ± 0.0064, respectively. The differences of *R*^2^ values from any two models are significant (p < 1e − 5, one-way ANOVA). Bonferroni correction was applied to control the family-wise error rate (“FWER”); since all comparisons showed p < 1e − 5, we could guarantee the probability of having one or more false positives is less 1e − 4. With this conservative Bonferroni correction, we conclude with confidence that the difference between any two models is significant. Figure 8 presents representative prediction results from the mechanism-based model and four machine learning models (Fig. 8 first three rows), as well as the scatter plot of prediction against measurement for each of the five models (Fig. 8 last row). The random forest model performed the best for both live and dead cells, regardless of whether the confluence was low, intermediate, or high. The mechanism-based model presented an intermediate performance compared to the other models, with higher error when the confluence was very low or very high.

**Table 6:** Comparison of performance between the mechanism-based and machine learning models.

Model score (*R*^2^)cs		Mechanism-based	Linear regression	KNN regression	Decision tree	Random forest
Training	Live	0.9750 ± 0.0000	0.7958 ± 0.0023	0.9179 ± 0.0017	1.0000 ± 0.0000	0.9994 ± 0.0000
	Dead	0.9504 ± 0.0000	0.6097 ± 0.0026	0.9387 ± 0.0014	1.0000 ± 0.0000	0.9988 ± 0.0000
	Total	0.9627 ± 0.0000	0.7027 ± 0.0019	0.9283 ± 0.0014	1.0000 ± 0.0000	0.9991 ± 0.0000
Validation	Live	0.8927 ± 0.0062	0.7843 ± 0.0079	0.8055 ± 0.0112	0.9146 ± 0.0042	0.9428 ± 0.0025
	Dead	0.6466 ± 0.0212	0.5963 ± 0.0080	0.8761 ± 0.0065	0.8638 ± 0.0062	0.9092 ± 0.0039
	Total	0.7696 ± 0.0130	0.6903 ± 0.0064	0.8408 ± 0.0075	0.8892 ± 0.0046	0.9260 ± 0.0028

**Figure 9:**
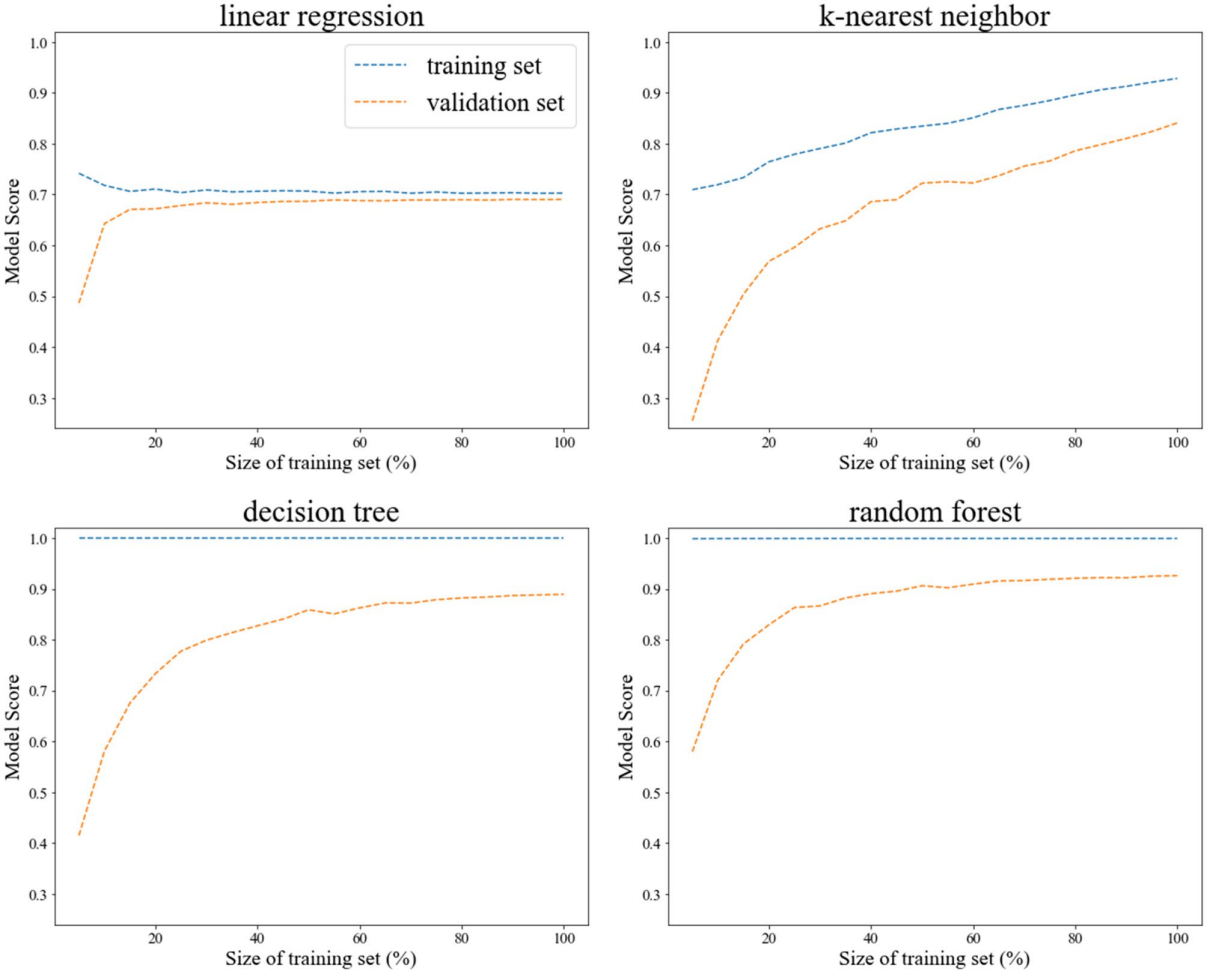
Learning curves of the machine learning models. For each panel, the results for the training and validation sets are shown for a particular model. The model score (*R*^2^) on the training set (blue curve) and validation set (orange curve) is plotted against the percentage of the complete training set.

Learning curves (Fig. 9) were used to evaluate the performance of machine learning and check for potential overfitting. When we increased the size of training set from 5 to 100% of the complete training set, the score on the validation set increased from 0.4863 ± 0.0849 to 0.6903 ± 0.0051 (+ 41.9%) for the Linear Regression model, from 0.2552 ± 0.0259 to 0.8408 ± 0.0061 (+ 229.5%) for the K-Nearest Neighbor regression model, from 0.4149 ± 0.0398 to 0.8892 ± 0.0037 (+ 114.3%) for the Decision Tree model, and from 0.5798 ± 0.0266 to 0.9260 ± 0.0023 (+ 59.7%) for the Random Forest model.

## Discussion

This study sought to extend the mathematical model we had previously developed^26^ that predicts tumor cell growth depending on the availability of glucose so we can apply it to investigate the effects of interventions by study design to perturb glucose accessibility. Specifically, we applied Cytochalasin B, an inhibitor for glucose uptake, as the treatment to investigate. We targeted the uptake of glucose in an extension of our model to avoid further complicating the system with the mechanism describing glucose consumption post-treatment. To achieve this goal, we proposed to model the total glucose level in the system and the accessible glucose level to the tumor cells. We assumed the consumption of glucose and all the metabolism-related tumor cell growth only depends on the intracellular glucose availability and not the total glucose level in the environment. Therefore, the accessible glucose level at time *t*, *G*_*acs*_(*t*), replaced the glucose level at time *t*, *G*(*t*), in the original system of coupled ODEs. As a result, we needed an additional equation to relate the accessible and total glucose levels which we achieved by introducing an inhibition constant depending on the dose of the inhibitor. In the resulting (extended) model (i.e., Eqs. (1)–(7)), the proliferation rate, the glucose depletion induced death rate, the consumption rate of glucose, and the newly introduced inhibition constant were considered as global parameters, while the death rate induced by the bystander effect (quantifying the effects of dead cells accumulated in the environment) was considered as a local parameter that would vary as a function of initial conditions. Time-resolved curves of tumor cell number for both live and dead tumor cells with different initial conditions, with or without treatment, were generated from time-resolved microscopy images for model calibration and validation. While we would expect the effect of Cytochalasin B to be stronger at lower extracellular glucose levels, this is not always clearly reflected in the confluence curves of treated tumor cells. Recall that the cell confluence is a net effect of both cell proliferation and death—with two sources (glucose depletion and bystander effect) contributing to cell death. When the extracellular glucose level is lower, there is (in general) more cell death (regardless of Cytochalasin B level) compared to high glucose levels and, therefore, more contribution from the third term on the right-hand side of Eq. (1). Thus, the effect of Cytochalasin B, although higher, is diluted by the contribution from the bystander effect when we compare the net change in the cell confluence. We investigated the relationship between the bystander effect induced death rate (*k*_*bys*_) and the initial glucose concentration. A negative correlation (partial correlation coefficient = − 0.41) between *k*_*bys*_ and the total initial glucose level was found. However, when we investigated the correlation between *k*_*bys*_ and the accessible glucose level, a stronger negative correlation (partial correlation coefficient = − 0.73) was found. This pattern matched our previous finding^26^ (i.e., a partial correlation coefficient of − 0.72 between *k*_*bys*_ and the initial glucose level), with the initial glucose level replaced by the initial accessible glucose level.

Numerous mathematical approaches have been developed to model tumor metabolism and growth, but few investigated a nutrient perturbation resulting from a mechanism-directed therapy; e.g., the application of an inhibitor of nutrient transport. In the present study, we not only extended our original model to account for the effect of mechanism-directed therapy, but also validated the model with experimental data obtained from time-resolved microscopy. To accomplish this, we introduced only one extra parameter to quantify the effect of the treatment, without the need for modeling complicated signaling pathways which would lead to a large set of free parameters. The approach for model calibration described in section "Calibration of the mechanism-based model with Dataset B to reduce the parameter space for Gin" reduced the potential range of the parameter values for *G*_*in*_, so we could obtain a more reliable estimate for the constant inhibition from the calibration described in section "Calibration of the mechanism-based model with Dataset B to estimate kbys and Gin". Importantly, by allowing the bystander effect induced death rate to be a local parameter the model had the flexibility to optimally describe the data. The resulting model was validated in a triple-negative breast cancer cell line (MDA-MB-231). With the model calibrated from the training data, we could predict tumor cell growth with our model with confidence, using the initial nutrient condition, seeding density, and dose of Cytochalasin B in the validation data. This experimental-mathematical approach presents a framework that enables one to test and quantify the effects of drugs designed to perturb intracellular glucose level in any number of cancer cell lines. In particular, we note that the original model was also tested in a HER2 + breast cancer cell line (BT-474)^26^.

We compared the performance of the mechanism-based model with four common machine learning models. The random forest model, featuring ensemble learning, presented the highest accuracy with the validation set. The decision tree model performed the best with the training set but not with the validation set, suggesting it may be overfitting. The linear regression model, with the least flexibility and lowest complexity, performed the worst in both the training and validation sets. The mechanism-based model presented an intermediate accuracy, with *R*^2^ lower than the *k*-nearest neighbor model and higher than the linear regression model. We further evaluated the performance of the machine learning models with learning curves. Though both decision tree and random forest models present seemingly unrealistic high performance on the training set, they do learn better from more data, and the gap between the training performance and validation performance is reduced and the validation performance is improved. With the complete training set, they become well generalized to make predictions on unseen data. In addition, the gap between the model performance on the training and validation sets of the Decision Tree (0.11) and Random Forest model (0.07) is between the KNN (0.09) and mechanism-base models (0.19). As neither the learning curves nor the training/validation performance comparison indicates these two models (i.e., decision tree and random forest models) are overfitting, their high scores on the training set do not seem to be a cause for concern.

Although the mechanism-based model did not beat the best machine learning models as quantified by the *R*^*2*^, it brings the important benefit of easy interpretation as it explicitly accounts for underlying biological mechanisms, entities not typically found in machine learning approaches^26^. Having a mechanism-based model provides guidance for further experiments and analyses that are not directly possible by interpreting the results from a machine learning approach. These studies include, for example, identifying optimal treatment protocols and experimental designs. It is possible that improved predictive accuracy could be achieved using more advanced techniques like feature selection, dimension reduction, or hyperparameter tuning to further enhance the model performance.

There are several opportunities to extend the results presented in this effort. As in our previous study^26^, we assume that the effect on glucose availability, as a result of both cell consumption and inhibition on glucose uptake, was entirely reflected by the time-resolved tumor cell number, through either growth or death. This could be an oversimplification, considering the complexity of the phenomenon. Therefore, we compared confluence time courses of tumor cells supplied with the highest glucose concentration and treated with Cytochalasin B, against tumor cells supplied with different glucose concentrations and no Cytochalasin B, to estimate the accessible glucose level in the treated cells. A high CCC suggested the glucose level in the treated cells was close to the glucose level in the cells supplied with a different glucose level but no treatment. While this comparison provides an indirect measurement of the glucose available in the treated cells, future studies should seek a direct measurement of the nutrient dynamics. In particular, we did not explicitly model the effect of Cytochalasin B as an inhibitor of actin polymerization^52^, but only as an inhibitor of glucose uptake. We simplified our model to be a function of accessible glucose level, driven by metabolism, where the effect of actin polymerization was implicitly captures as “glucose depletion”. This could be an oversimplification resulting in an overestimation of the effect of glucose metabolism and would require further studies to separate the effect of Cytochalasin B’s dual role. Additionally, we only tested two doses of Cytochalasin B and would require additional measurements to confirm a relationship between the inhibition constant and the dose. Knowing such a relationship would enable the model to perform predictions at doses not yet tested. Another area for improvement would be to employ time-resolved, non-invasive imaging to track the nutrient level in the cells; we have attempted to develop such a technique based on FRET reporters transfected into the MDA-MB-231 cell line^53^, but it only provides a relative measure of glucose and lactate concentrations. The absolute measurement of the dynamics of lactate concentration would also allow us to include more details in tumor cell metabolism in the model.

## Conclusion

We have developed a mechanism-based model that predicts how glucose accessibility influences tumor cell growth by accounting for the effect of metabolism-directed therapy. The model was calibrated and validated in a triple-negative breast cancer cell line. We were able to quantify the dose-dependent inhibition constant and confirm that the dependence of the bystander effect death rate on the initial glucose level was highly correlated to the accessible glucose level. This mechanism-based model presents predictive capability comparable to complicated machine learning models, but is much easier to interpret and provides the opportunity to directly guide further experiments and analysis in a way not possible with a machine learning approach.

## Supplementary Information

Supplementary Information.

## Data Availability

The datasets used and/or analyzed during the current study available from the corresponding author on reasonable request.

## References

[1] Hanahan D, Weinberg RA (2011). Hallmarks of cancer: The next generation. Cell.

[2] El Mjiyad N, Caro-Maldonado A, Ramírez-Peinado S, Muñoz-Pinedo C (2010). Sugar-free approaches to cancer cell killing. Oncogene.

[3] Kennedy, K. M. & Dewhirst, M. W. Tumor metabolism of lactate: The influence and therapeutic potential for MCT and CD147 regulation. 10.2217/fon.09.145 (2009). 10.2217/fon.09.145 PMC2819205 20021214

[4] Tennant DA, Durán RV, Gottlieb E (2010). Targeting metabolic transformation for cancer therapy. Nat. Rev. Cancer.

[5] Vander Heiden MG (2011). Targeting cancer metabolism: A therapeutic window opens. Nat. Rev. Drug Discov..

[6] Anderson ARA, Quaranta V (2008). Integrative mathematical oncology. Nat. Rev. Cancer.

[7] Yankeelov TE, Atuegwu NC, Deane NG, Gore JC (2010). Modeling tumor growth and treatment response based on quantitative imaging data. Integr. Biol..

[8] Yankeelov TE (2013). Clinically relevant modeling of tumor growth and treatment response. Sci. Transl. Med..

[9] Enderling H, Chaplain M (2014). Mathematical modeling of tumor growth and treatment. CPD.

[10] Yankeelov TE, Quaranta V, Evans KJ, Rericha EC (2015). Toward a science of tumor forecasting for clinical oncology. Can. Res..

[11] Yankeelov TE (2016). Multi-scale modeling in clinical oncology: Opportunities and barriers to success. Ann. Biomed. Eng..

[12] Szymańska Z, Cytowski M, Mitchell E, Macnamara CK, Chaplain MAJ (2018). Computational modelling of cancer development and growth: Modelling at multiple scales and multiscale modelling. Bull. Math. Biol..

[13] Anderson ARA, Maini PK (2018). Mathematical oncology. Bull. Math. Biol..

[14] Rockne RC (2019). The 2019 mathematical oncology roadmap. Phys. Biol..

[15] Bull JA, Byrne HM (2022). The hallmarks of mathematical oncology. Proc. IEEE.

[16] Mendoza-Juez B, Martínez-González A, Calvo GF, Pérez-García VM (2012). A mathematical model for the glucose-lactate metabolism of in vitro cancer cells. Bull. Math. Biol..

[17] Astanin S, Preziosi L (2009). Mathematical modelling of the Warburg effect in tumour cords. J. Theor. Biol..

[18] McGillen JB (2014). Glucose–lactate metabolic cooperation in cancer: Insights from a spatial mathematical model and implications for targeted therapy. J. Theor. Biol..

[19] Phipps C, Molavian H, Kohandel M (2015). A microscale mathematical model for metabolic symbiosis: Investigating the effects of metabolic inhibition on ATP turnover in tumors. J. Theor. Biol..

[20] Robertson-Tessi M, Gillies RJ, Gatenby RA, Anderson ARA (2015). Impact of metabolic heterogeneity on tumor growth, invasion, and treatment outcomes. Can. Res..

[21] Anderson ARA, Weaver AM, Cummings PT, Quaranta V (2006). Tumor morphology and phenotypic evolution driven by selective pressure from the microenvironment. Cell.

[22] Chen Y, Wang H, Zhang J, Chen K, Li Y (2016). Simulation of avascular tumor growth by agent-based game model involving phenotype–phenotype interactions. Sci. Rep..

[23] Kianercy A, Veltri R, Pienta KJ (2014). Critical transitions in a game theoretic model of tumour metabolism. Interface Focus.

[24] Archetti M (2015). Heterogeneity and proliferation of invasive cancer subclones in game theory models of the Warburg effect. Cell Prolif..

[25] Epstein T, Gatenby RA, Brown JS (2017). The Warburg effect as an adaptation of cancer cells to rapid fluctuations in energy demand. PLoS ONE.

[26] Yang J (2021). An experimental-mathematical approach to predict tumor cell growth as a function of glucose availability in breast cancer cell lines. PLoS ONE.

[27] Cui S (2002). Analysis of a mathematical model for the growth of tumors under the action of external inhibitors. J. Math. Biol..

[28] Roy M, Finley SD (2017). Computational model predicts the effects of targeting cellular metabolism in pancreatic cancer. Front. Physiol..

[29] Eyassu F, Angione C (2017). Modelling pyruvate dehydrogenase under hypoxia and its role in cancer metabolism. R. Soc. Open Sci..

[30] Katzir R (2019). The landscape of tiered regulation of breast cancer cell metabolism. Sci. Rep..

[31] Kim B-C (2021). Machine learning model for lymph node metastasis prediction in breast cancer using random forest algorithm and mitochondrial metabolism hub genes. Appl. Sci..

[32] Gómez OV (2022). Analysis of cross-combinations of feature selection and machine-learning classification methods based on [18F]F-FDG PET/CT radiomic features for metabolic response prediction of metastatic breast cancer lesions. Cancers.

[33] SOP: Thawing, propagation and cryopreservation of NCI-PBCF-HTB26 (MDA-MB-231). 25.

[34] Estensen RD, Plagemann PGW (1972). Cytochalasin B: Inhibition of glucose and glucosamine transport. Proc. Natl. Acad. Sci. USA.

[35] Freeman SM, Abboud CN, Whartenby KA, Packman CH, Koeplin DS, Moolten FL, Abraham GN (1993). The "bystander effect": tumor regression when a fraction of the tumor mass is genetically modified. Cancer Res..

[36] Li Bi W, Parysek LM, Warnick R, Stambrook PJ (1993). In vitro evidence that metabolic cooperation is responsible for the bystander effect observed with HSV tk retroviral gene therapy. Hum. Gene Ther..

[37] Maulud D, Abdulazeez AM (2020). A review on linear regression comprehensive in machine learning. JASTT.

[38] Fix E, Hodges JL (1989). Discriminatory analysis. Nonparametric discrimination: Consistency properties. Int. Stat. Rev..

[39] Altman NS (1992). An introduction to kernel and nearest-neighbor nonparametric regression. Am. Stat..

[40] Loh W (2011). Classification and regression trees. WIREs Data Min. Knowl. Discov..

[41] Ho, T. K. Random decision forests. In *Proceedings of 3rd International Conference on Document Analysis and Recognition* vol. 1 278–282 (IEEE Comput. Soc. Press, 1995).

[42] Ho TK (1998). The random subspace method for constructing decision forests. IEEE Trans. Pattern Anal. Mach. Intell..

[43] Opitz D, Maclin R (1999). Popular ensemble methods: An empirical study. Jair.

[44] Polikar R (2006). Ensemble based systems in decision making. IEEE Circuits Syst. Mag..

[45] Rokach L (2010). Ensemble-based classifiers. Artif. Intell. Rev..

[46] Breiman L (1996). Bagging predictors. Mach. Learn..

[47] Kleinberg EM (1990). Stochastic discrimination. Ann. Math. Artif. Intell..

[48] Kleinberg EM (1996). An overtraining-resistant stochastic modeling method for pattern recognition. Ann. Stat..

[49] Kleinberg EM (2000). On the algorithmic implementation of stochastic discrimination. IEEE Trans. Pattern Anal. Mach. Intell..

[50] Friedman JH (2017). The Elements of Statistical Learning: Data Mining, Inference, and Prediction.

[51] Saravanan N, Sathish G, Balajee JM (2018). Data wrangling and data leakage in machine learning for healthcare. JETIR.

[52] MacLean-Fletcher S (1980). Mechanism of action of cytochalasin B on actin. Cell.

[53] Yang J (2021). Longitudinal FRET imaging of glucose and lactate dynamics and response to therapy in breast cancer cells. Mol. Imaging Biol..

