# Supplementary material for: Comparing mechanism-based and machine learning models for predicting the effects of glucose accessibility on tumor cell proliferation
Source: Sci Rep. 2023 Jun 27;13:10387. doi: 10.1038/s41598-023-37238-2 (PMC10300192; doi:10.1038/s41598-023-37238-2)
Supplement: Supplementary file 1 — Supplementary Information. [file 41598_2023_37238_MOESM1_ESM.docx]

| Evaluation variables | Corresponding computation |
| --- | --- |
| Mean percent error |  |
| Percent error at end of experiment |  |
| Mean error |  |
| Error at end of experiment |  |

**Table S1**. The table presents the equations used to evaluate the model’s (i.e., Eqs. [1] - [7]) performance, where *X_model,ij_* is the number of live or dead cells of well *j* at timepoint *i* calculated from the model, *X_data,ij_* is the number of live or dead cells of well *j* at timepoint *i* from the measured data, *t* is the total number of timepoints, *w* is the total number of wells, and *t_end_* is the last timepoint at the end of experiment. These errors were calculated from model outputs and experimental measurements to evaluate the model performance across the whole experiment or at the end of the experiment. Results are shown in Tables 3 - 5 and reported in Sections 3.3.3 and 3.4.


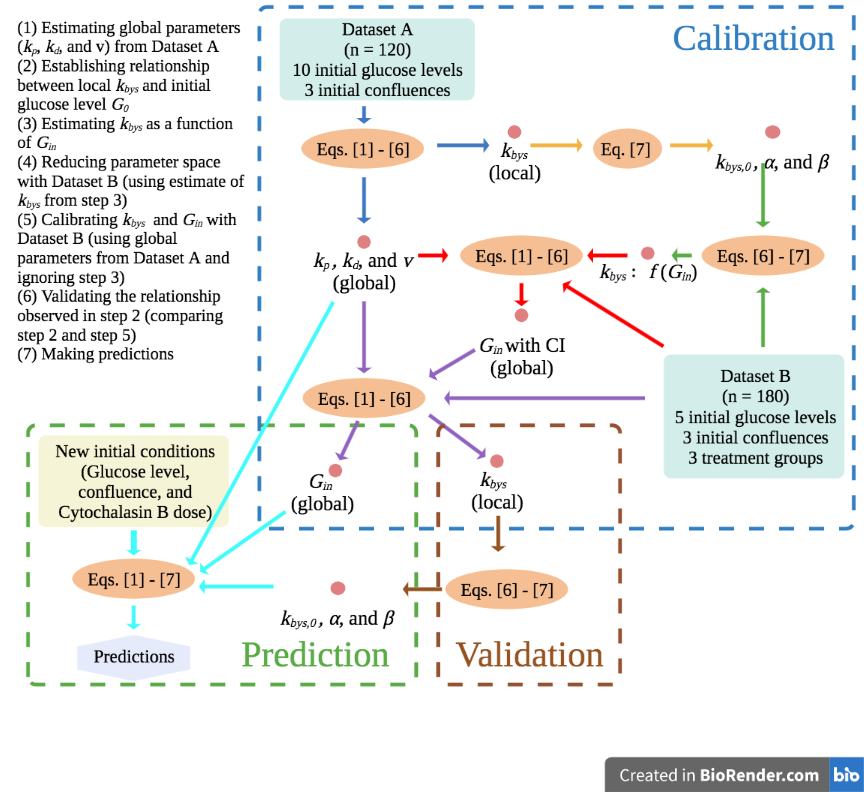


**Figure S1. Detailed flow chart of the calibration/prediction process.** The figure shows the dataflow throughout the calibration and prediction steps, where data are shown in light green boxes, parameters are shown as red dots, and models (i.e., the equations involved) are shown in orange ellipses. Initial conditions used for prediction are shown in a beige box, while predicted time courses of confluence for the tumor cells are shown in a light blue hexagon. During each step, data (complete time courses or conditions) and parameter(s) are input to estimate the unknown parameters within a model. Each step is marked with arrows of the same color, where blue, orange, green, red, purple, brown, and cyan arrows represent steps 1 – 7, respectively. These “steps” are referred to frequently in the Materials and Methods section.
